# Reversal of Obesity and Insulin Resistance by a Non-Peptidic Glucagon-Like Peptide-1 Receptor Agonist in Diet-Induced Obese Mice

**DOI:** 10.1371/journal.pone.0014205

**Published:** 2010-12-03

**Authors:** Min He, Haoran Su, Weiwei Gao, Stina M. Johansson, Qing Liu, Xiaoyan Wu, Jiayu Liao, Andrew A. Young, Tamas Bartfai, Ming-Wei Wang

**Affiliations:** 1 The National Center for Drug Screening, Shanghai, China; 2 The State Key Laboratory of Drug Research, Shanghai Institute of Materia Medica, Chinese Academy of Sciences, Shanghai, China; 3 Molecular and Integrative Neurosciences Department, The Scripps Research Institute, La Jolla, California, United States of America; University of Bremen, Germany

## Abstract

**Background:**

Glucagon-like peptide-1 (GLP-1) is recognized as an important regulator of glucose homeostasis. Efforts to utilize GLP-1 mimetics in the treatment of diabetes have yielded clinical benefits. A major hurdle for an effective oral therapy has been the difficulty of finding a non-peptidic GLP-1 receptor (GLP-1R) agonist. While its oral bioavailability still poses significant challenges, Boc5, one of the first such compounds, has demonstrated the attainment of GLP-1R agonism in diabetic mice. The present work was to investigate whether subchronic Boc5 treatment can restore glycemic control and induce sustainable weight loss in diet-induced obese (DIO) mice, an animal model of human obesity and insulin resistance.

**Methodology/Principal Findings:**

DIO mice were treated three times a week with Boc5 (0.3, 1 and 3 mg) for 12 weeks. Body weight, body mass index (BMI), food intake, fasting glucose, intraperitoneal glucose tolerance and insulin induced glucose clearance were monitored regularly throughout the treatment. Glucose-stimulated insulin secretion, β-cell mass, islet size, body composition, serum metabolic profiles, lipogenesis, lipolysis, adipose hypertrophy and lipid deposition in the liver and muscle were also measured after 12 weeks of dosing. Boc5 dose-dependently reduced body weight, BMI and food intake in DIO mice. These changes were associated with significant decreases in fat mass, adipocyte hypertrophy and peripheral tissue lipid accumulation. Boc5 treatment also restored glycemic control through marked improvement of insulin sensitivity and normalization of β-cell mass. Administration of Boc5 (3 mg) reduced basal but enhanced insulin-mediated glucose incorporation and noradrenaline-stimulated lipolysis in isolated adipocytes from obese mice. Furthermore, circulating leptin, adiponectin, triglyceride, total cholesterol, nonesterified fatty acid and high-density lipoprotein/low-density lipoprotein ratio were normalized to various extents by Boc5 treatment.

**Conclusions/Significance:**

Boc5 may produce metabolic benefits via multiple synergistic mechanisms and may represent an attractive tool for therapeutic intervention of obesity and diabetes, by means of non-peptidic GLP-1R agonism.

## Introduction

In the past decades, obesity has become a worldwide epidemic due to excessive energy intake and lack of physical exercises [1], [2]. Associated with obesity, metabolic disorders including hyperinsulinemia, impaired glucose tolerance and dyslipidemia are often observed, which increase the risk for type 2 diabetes mellitus (T2DM), cancer and heart diseases [3]–[7]. Although diet control and life style modification remain the first steps in obesity management [8], [9], the use of pharmaceutical agents may sometimes be indispensable for long-term treatment of obesity [10].

Gastrointestinal hormones secreted in response to nutrient ingestion play essential roles at multiple levels in the regulation of energy homeostasis [11], and have been regarded as potential therapeutic targets for safe and sustainable weight loss [12]. Glucagon-like peptide-1 (GLP-1), an insulinotropic gastrointestinal peptide produced mainly from intestinal endocrine L-cells, inhibits glucagon secretion, stimulates glucose-dependent insulin production, improves insulin sensitivity, delays gastric emptying as well as increases satiety [13]–[16]. Peptidic GLP-1 receptor (GLP-1R) agonists, exemplified by the first incretin mimetic, Exendin-4 (Exenatide), exert many of the glucose regulatory actions observed with GLP-1 [17], thereby possessing favorable effects in the treatment of T2DM [18]. In addition to the benefits in glycemic control, chronic treatment of GLP-1 analogues was also capable of inducing significant weight loss in rodents or patients with T2DM [18], [19]. Hence, GLP-1R agonists represent a promising class of new drugs with dual anti-obesity and anti-diabetic properties [20].

The approach to elevate endogenous GLP-1 levels by inhibition of the predominant GLP-1 degrading enzyme, dipeptidyl peptidase-IV (DPP-IV), has been proven useful for T2DM treatment [21], but does not seem to fully capture the anti-diabetic potential of GLP-1R agonism [22] in terms of promoting weight loss [23]. All the GLP-1R agonists developed to date, or currently under development, are of peptidic nature and this imposes certain limitations on their administration. Thus, there is considerable interest in the development of non-peptidic GLP-1R agonists [24], [25].

We have previously identified a substituted cyclobutane, Boc5, that acts *in vitro* and *in vivo* as a full GLP-1R agonist [26]. Boc5 is a small molecule compound with reasonable affinity for GLP-1R and good safety profile *in vivo*. It could simultaneously activate a broad spectrum of anti-diabetic effects including decline of blood glucose, inhibition of food intake, slowing of gastric emptying, stimulation of insulin secretion, elevation of insulin sensitivity and reduction of body weight in diabetic *db/db* mice [27]. Nevertheless, the *db/db* mice, as a leptin receptor-deficient rodent model, are unable to fully represent the pathogenesis of human obesity/diabetes. Many studies have used high fat diet (HFD) fed rodents to recapitulate the polygenic features of obesity that mimic human consumption patterns. This diet-induced obesity (DIO) model has been shown to be most efficient in C57BL/6J (C57) mice compared with other strains [28], [29]. When fed HFD, C57 mice are characteristic of overweight, hyperglycemia, hyperinsulinemia, glucose intolerance as well as dyslipidemia [30]. In the present study, we investigated a variety of metabolic consequences following subchronic Boc5 treatment of DIO mice to explore the potential therapeutic utility of this new class of GLP-1 mimetics.

## Results

### Effect on body weight

**Figure 1:**
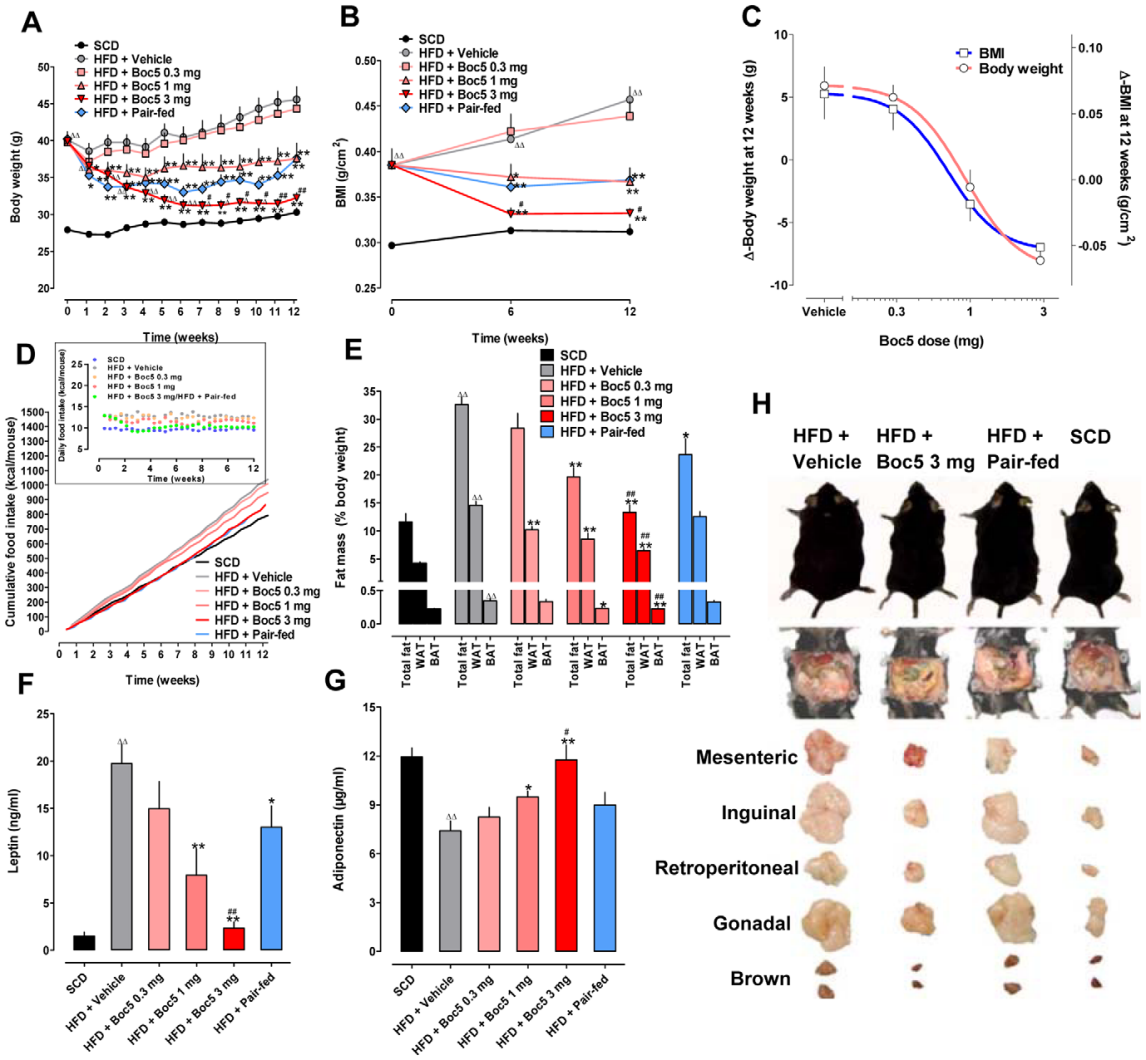
Effects of Boc5 on body weight, BMI, food intake, adiposity and circulating adipocytokine concentrations. (A) Time course of the effect on body weight (n = 16 per group). (B) BMI monitored before, during and after the treatment (n = 6 per group). (C) Dose-response profiles for weight (Δ-body weight) and BMI (Δ-BMI) changes over the 12-week period (n = 16 per group for weight and n = 6 per group for BMI). (D) Time course of the effect on cumulative and daily food intake (insert) (n = 16 per group). (E) Dose-dependent effects on whole fat mass (n = 6 per group), white and brown adipose tissues (WAT and BAT) as percentage of body weight (n = 8–14 per group). WAT were represented by mesenteric, inguinal, retroperitoneal and gonadal fat pads. (F) Serum leptin and (G) adiponectin levels measured at the end of the treatment (n = 9 for SCD and HFD groups; n = 6 for Boc5-treated groups). (H) Effects on the gross appearance of body shape (upper panel), abdominal fat (middle panel) and fat depots (lower panel) recorded at autopsy. Values represent mean±SEM. ^Δ^ *P*<0.05 and ^ΔΔ^ *P*<0.01 compared with SCD group; ^*^ *P*<0.05 and ^**^ *P*<0.01 compared with HFD group; ^#^ *P*<0.05 and ^##^ *P*<0.01 compared with pair-fed group that received an equal amount of food as 3 mg Boc5-treated mice.

Before initiation of Boc5 treatment, C57 mice were fed HFD for 12 weeks and only those that reached a body weight of ≥40 g and body mass index (BMI) of ≥0.39 g/cm^2^ [45.5% and 30.0% more than that of standard chow diet (SCD) controls, respectively] were selected and randomly distributed to each study group (Figures 1A and 1B). Intermittent Boc5 administration (3 times per week, tiw) led to a dose-dependent and significant reduction in body weight and BMI, which sustained over the entire treatment period (12 weeks). The mice ultimately exhibited 8.0 g (1 mg dose) to 13.3 g (3 mg dose) weight loss, or approximately 17.6% to 29.2% reduction from the level seen in vehicle-treated obese controls (45.6 g in weight); this was accompanied by a consistent and parallel decrease in BMI measurements (*P*<0.0001; Figures 1A and 1B). The corresponding ED_50_ values estimated were 0.9 mg for weight loss and 0.7 mg for BMI decrease, respectively (Figure 1C). For pair-fed mice constrained to a food intake equivalent to that of the 3 mg Boc5 treatment group, although a similar weight and BMI changes were observed, the effect was less pronounced than the Boc5-treated counterparts (*P*<0.01; Figures 1A and 1B). While Boc5 dose-dependently (*P*<0.0001) induced weight loss and BMI reduction in DIO mice, only the 3 mg dose produced a weight loss indistinguishable from SCD controls (*P* = 0.0684 for body weight and *P* = 0.0765 for BMI; Figures 1A and 1B). Similar to our previous findings in *db/db* mice, Boc5 dose-dependently inhibited cumulative food intake by up to 17% (approximately 11.5% of daily food intake) throughout the 12-week treatment course, and Boc5-treated mice (3 mg) ingested nearly the same amount of food as mice fed SCD (Figure 1D).

Effects of subchronic Boc5 treatment on body composition were examined in follow-up experiments. The brown fat and four white fat (mesenteric, inguinal, retroperitoneal and gonadal) depots sampled were about 1.2- to 3.4-fold heavier as a percentage of body weight in HFD mice than that of SCD controls prior to the treatment (Figure S1). When normalized for body weight after 12-week Boc5 injection, the weights of white fat pads, as well as brown fat pad (1 and 3 mg groups), were significantly reduced (*P*<0.0001, *P* = 0.0164 and *P* = 0.0022, respectively) (Figure 1E). Total fat analysis indicated that Boc5 treatment proportionately brought the body composition profile of obese mice close to that of lean animals (3 mg Boc5 group became indistinguishable, *P* = 0.41). The 10.3 g loss of total fat mass following 3 mg Boc5 treatment, calculated from the percentage of body weight, accounted for 77% of the 13.3 g weight reduction, relative to obese controls (Figure 1E). Notably, although pair-fed mice revealed obvious downward trend of total fat mass, the effect failed to reach the same level of the 3 mg Boc5 dose group.

The serum levels of leptin and adiponectin, two important adipocytokines secreted from white adipose tissue (WAT), were also altered after Boc5 treatment. Circulating leptin concentration in DIO mice was significantly higher than that in SCD controls (19.7 ng/ml *vs*. 1.5 ng/ml, *P*<0.0001). In contrast, Boc5 (3 mg) administration significantly (*P*<0.0001) reduced serum leptin level to a near normal value of 2.3 ng/ml (Figure 1F). Figure 1G shows that, prolonged exposure to HFD induced a 38% lower serum adiponectin level in DIO than in lean mice (*P* = 0.0004), whereas this level was remarkably increased (*P*<0.0001) by Boc5 to the normal range observed in SCD fed controls (11.7 µg/ml in 3 mg group *vs*. 11.9 µg/ml in SCD group). Although pair-fed mice displayed certain degree of improved circulating leptin and adiponectin concentrations, the effects were relatively marginal and not as pronounced as seen in 3 mg Boc5-treated animals (*P* = 0.0003 for leptin and *P* = 0.0444 for adiponectin; Figures 1F and 1G).

### Effect on adipocyte morphology

**Figure 2:**
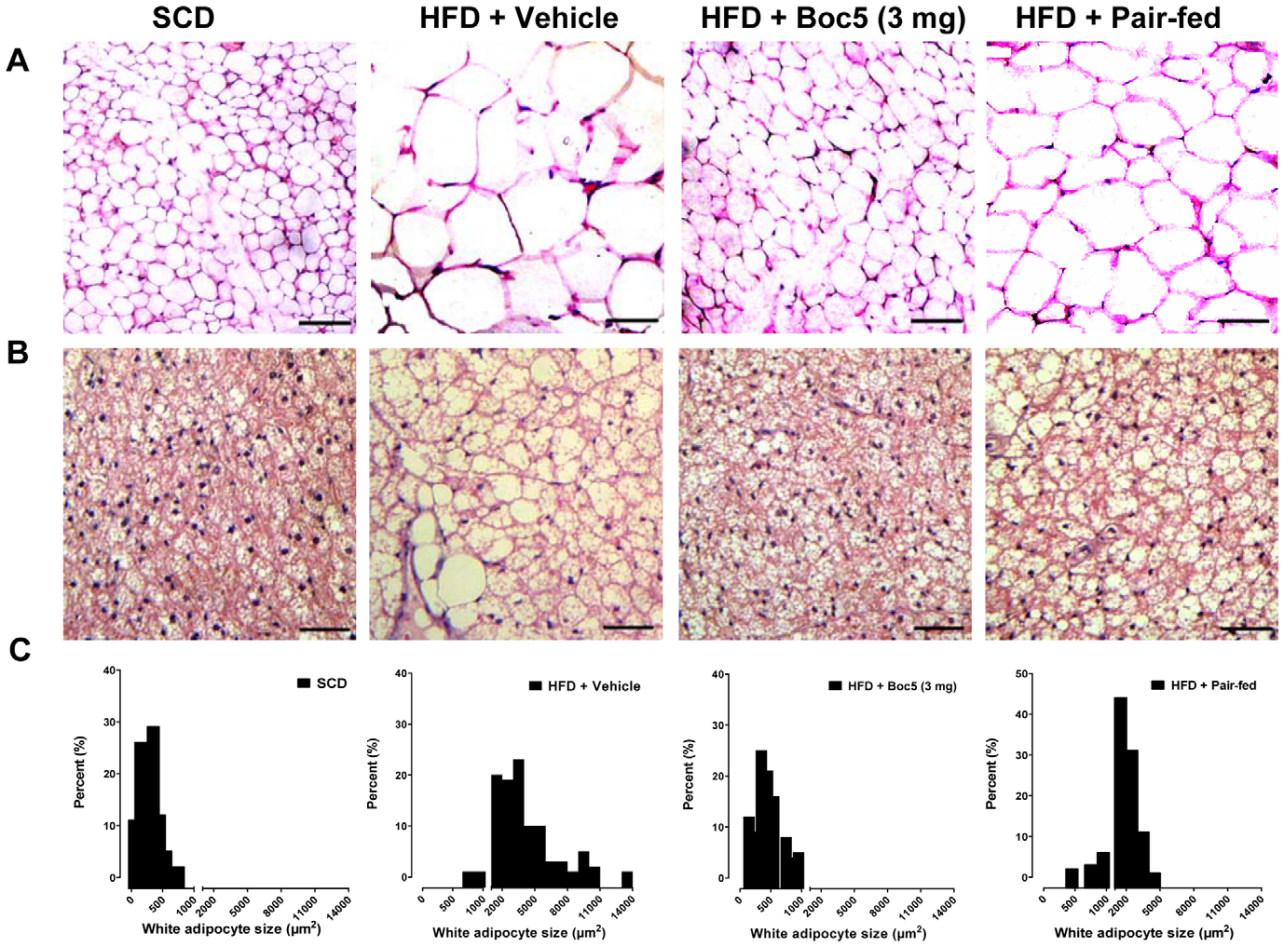
Effects of Boc5 on adipocyte morphology. Histological analysis of inguinal white (A) and brown (B) adipocytes isolated from lean (SCD) and obese (HFD) mice that received vehicle, Boc5 (3 mg) or pair-fed treatment (n = 3 per group). Sections (5 µm) were stained with H&E and representative images (×200 original magnification) obtained at the end of the 12-week observation period. (C) Distribution of the white adipocyte cell size. The mean surface area and the frequency distribution were calculated based on at least 500 cells from each mouse. Scale bars = 50 µm.

Histological analysis revealed that mice on HFD had larger white (inguinal) adipocytes and an increased accumulation of lipids in brown adipocytes relative to SCD controls; Boc5 treatment led to a substantial decrease in the size of adipocytes in inguinal WAT (Figure 2A); similar results were also observed in brown adipose tissue (BAT). Brown fat in vehicle-treated obese mice resembled white fat with a few massive lipid droplets invading in each brown adipocyte, while Boc5 treatment reversed such lipid deposition via replacing large lipid droplets with multiple small ones (Figure 2B). Morphometric analysis of adipocyte distribution along with their sizes indicated that Boc5 increased the population of small-sized adipocytes and decreased the population of large-sized adipocytes in WAT. As shown in Figure 2C, the size of white adipocytes in the 3 mg Boc5-treated group (466±21 µm^2^) was dramatically smaller than that in vehicle-treated controls (3884±249 µm^2^) and reached the adjacent level of SCD fed lean mice (292±15 µm^2^). In contrast, although the fat cell size in pair-fed mice became smaller and was equivalent to about 50% (1964±80 µm^2^) of obese mice, it was still obviously larger than that of 3 mg Boc5-treated animals (Figures 2A and 2C).

### Effects on glucose uptake and lipolysis

**Figure 3:**
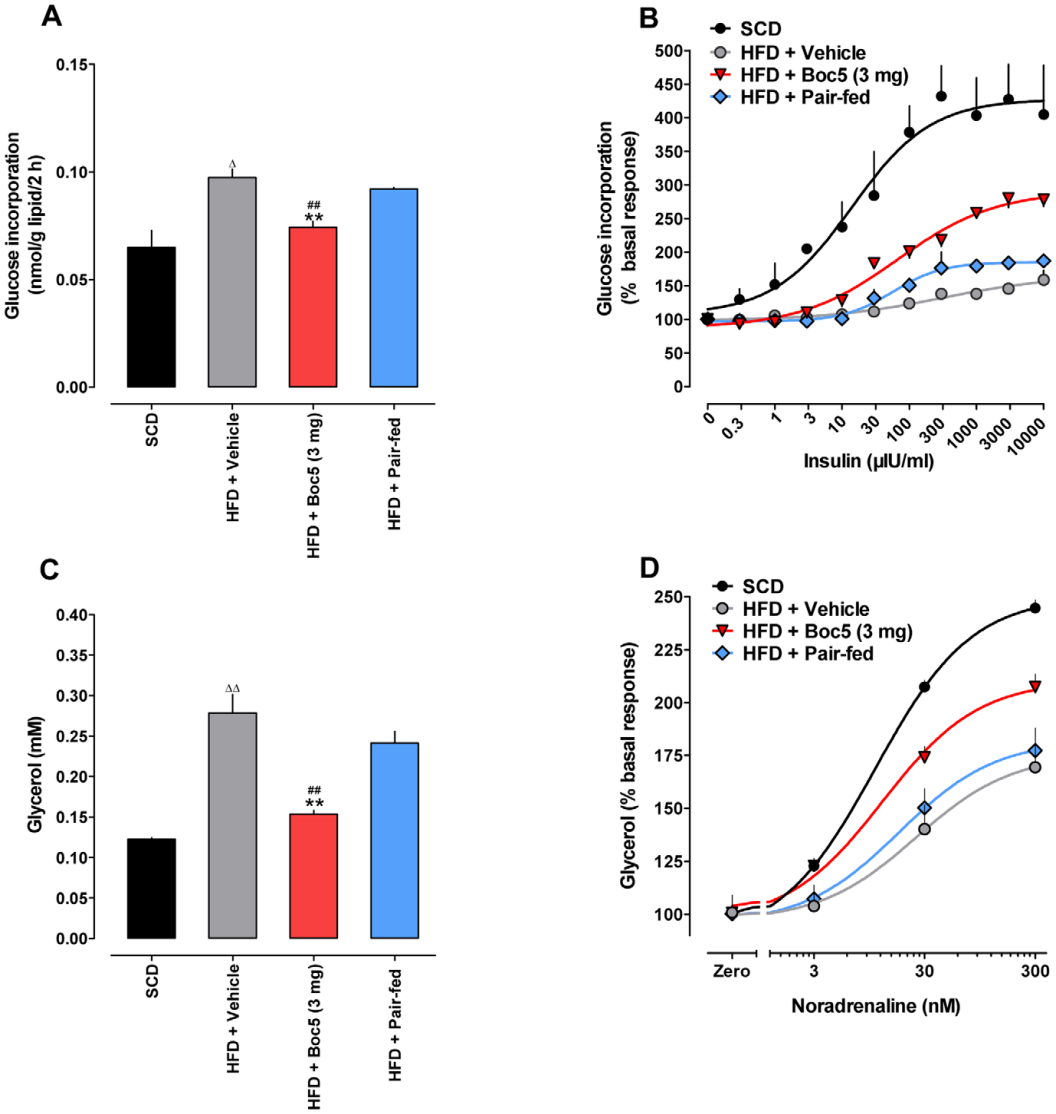
Effects of Boc5 on glucose uptake and lipolysis in isolated adipocytes. (A) Basal glucose uptake in the absence of insulin after 2 h of incubation. Data shown are the relative quantities of intracellular [3-^3^H]glucose normalized by the total lipid content. (B) Insulin-stimulated glucose incorporation expressed as a percentage of the basal response. (C) Basal lipolysis in the absence of noradrenaline after 1 h of incubation. Data shown are the absolute concentrations of glycerol resulted from triglyceride hydrolysis. (D) Noradrenaline-stimulated glycerol release expressed as a percentage of the basal response. Values represent mean±SEM of two to five independent *ex vivo* studies using adipocytes isolated from two (glucose uptake) or four (lipolysis) mice for each experiment. ^Δ^ *P*<0.05 and ^ΔΔ^ *P*<0.01 compared with SCD group; ^**^ *P*<0.01 compared with HFD group; ^##^ *P*<0.01 compared with pair-fed group that received an equal amount of food as 3 mg Boc5-treated mice.

To investigate whether lipid metabolism was modified after subchronic Boc5 therapy, *ex vivo* experiments were carried out using the adipocytes isolated from treated mice. We first examined the glucose uptake capacity of gonadal adipocytes from four different treatment groups (SCD, HFD, 3 mg of Boc5 and pair-fed) by measuring the incorporation of D-[3-^3^H]glucose into lipids, as an index of lipogenesis. As shown in Figure 3A, the value of basal glucose incorporation was significantly increased in obese mice compared with that in lean controls (*P* = 0.0213). Boc5 treatment, but not pair-feeding, was able to reduced this high basal glucose uptake (*P* = 0.0082) towards the normal level (*P* = 0.3289). It follows that the action of insulin to stimulate glucose incorporation was markedly attenuated in adipocytes of DIO mice (Figure 3B), that was partially reversed by Boc5 treatment (increased by 1.8-fold compared to obese controls), with an estimated EC_50_ value for insulin of 67.3 µIU/ml (315.0 µIU/ml in untreated animals), thereby pointing to a significant improvement of insulin sensitivity. Although insulin-dependent glucose incorporation was also partially restored in pair-fed controls (Figure 3B), the effect was much less pronounced than in their Boc5-treated counterparts.

We also assayed the levels of basal and L-noradrenaline (NA)-stimulated glycerol released to the culture medium from isolated adipocytes as an indicator of triglyceride metabolism. As shown in Figure 3C, basal lipolysis in DIO mice was significantly higher than that in lean animals (0.28 mM and 0.12 mM, respectively, *P*<0.0001), whereas Boc5, rather than the pair-fed control, led to a 46.4% decrease in basal glycerol release, albeit not completely normalized (*P*<0.0001). The concentrations of NA (3, 30 and 300 nM) used in this study were chosen according to the literature [31], which indeed caused dose-dependent increases (ED_50_ = 11.3 nM) of lipolysis in SCD fed mice (Figure 3D). In the obese group, however, NA-stimulated lipolytic activity was significantly suppressed (ED_50_ = 26.5 nM); Boc5 treatment resulted in an evident improvement (ED_50_ = 13.2 nM), while lipolytic response to NA in pair-fed mice was only minimally recovered (ED_50_ = 18.6 nM; Figure 3D).

### Effects on glycemic control and insulin sensitivity

High fat feeding induced mild hyperglycemia in obese mice compared to those received a regular chow diet, with basal fasting glucose levels ranging from 4.4±0.4 mM (lean) to 8.0±2.1 mM (obese) (*P* = 0.0012; Table 1). The latter value was progressively worsened to around 12 mM over the 12-week observation period in DIO mice treated with vehicle or low doses of Boc5 (0.3 and 1 mg). In contrast, the 3 mg dose led to a significant decrease in fasting glucose (8.3±0.9 mM, *P*<0.0001) to the comparable level seen in lean animals (7.1±1.5 mM, *P* = 0.1260). Nevertheless, the effect of pair-feeding was only marginal (11.3±1.9 mM, *P* = 0.5771 *vs.* obese mice; Table 1).

**Table 1:** Fasting blood glucose levels (mM) measured before and during Boc5 treatment.

		HFD				
Weeks	SCD	Vehicle	Boc5 0.3 mg	Boc5 1 mg	Boc5 3 mg	Pair-fed
**0**	4.4±0.4	8.0±2.1ΔΔ	8.0±2.1	8.0±2.1	8.0±2.1	8.0±2.1
**3**	5.8±1.0	9.0±1.9ΔΔ	8.2±1.3	9.7±2.1	7.5±1.0#	9.2±0.8
**6**	5.7±0.6	9.5±1.5ΔΔ	8.2±1.3	10.6±0.4	8.0±1.2*, #	11.2±2.2
**9**	6.4±1.0	11.0±0.7ΔΔ	11.3±1.4	11.2±2.2	9.5±0.6**, #	11.4±1.7
**12**	7.1±1.5	12.1±2.4ΔΔ	12.0±0.9	11.9±1.3	8.3±0.9**, ##	11.3±1.9

Values represent mean±SEM (n = 6–10 per group).

ΔΔ *P*<0.01 *vs*. normal diet (SCD) group;

*P<0.05 and

**P<0.01 *vs*. high fat diet (HFD) group that received vehicle treatment;

# *P*<0.05 and

## *P*<0.01 *vs*. HFD group that was pair-fed an equal amount of food as 3 mg Boc5-treated mice.

**Figure 4:**
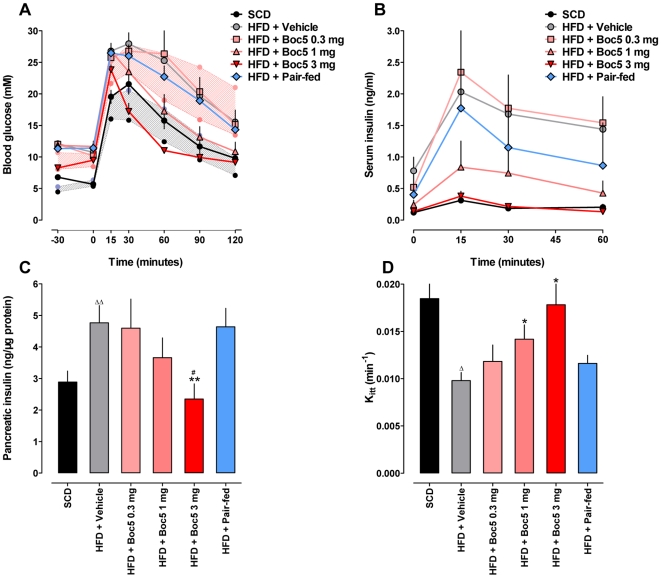
Effects of Boc5 on glucose homeostasis and insulin sensitivity. (A) Intraperitoneal glucose tolerance test (IPGTT) after 12 weeks of Boc5 treatment in diet-induced obese (DIO) mice (n = 6–8 per group). The pink and gray bands respectively denote mean±SD of blood glucose levels measured in obese (HFD) and lean (SCD) mice prior to treatment. The glucose area-under-curve integrated from 0–120 min (AUC_120_) was calculated for each mouse. (B) Serum insulin response to an intraperitoneal glucose challenge one day after the cessation of Boc5 therapy. The insulin area-under-curve integrated from 0–60 min (AUC_60_) was calculated for each mouse (n = 8 per group). (C) Pancreatic insulin content measured at the end of treatment (n = 6–9 per group). (D) Effect on K_itt_, a measure of insulin-mediated glucose clearance, in each treatment group (n = 8 per group). Values represent mean±SEM. ^Δ^ *P*<0.05 and ^ΔΔ^ *P*<0.01 compared with SCD group; ^*^ *P*<0.05 and ^**^ *P*<0.01 compared with HFD group; ^#^ *P*<0.05 compared with pair-fed group that received an equal amount of food as 3 mg Boc5-treated mice.

Glucose tolerance was quantified as the area-under-curve integrated from 0–120 min (AUC_120_) after an intraperitoneal glucose tolerance test (IPGTT). Prior to initiating therapy with Boc5, DIO mice showed impaired glucose tolerance relative to SCD controls (*P*<0.0001; Figure 4A, Figure S2), it was progressively improved during the course of Boc5 treatment (Figure S2). An IPGTT conducted at the end of 12-week Boc5 therapy revealed a dose-dependent restoration (*P*<0.0001) of glucose tolerance, such that the glucose profile of mice receiving 1 and 3 mg Boc5 was indistinguishable from that of SCD fed mice (*P* = 0.0626 and *P* = 0.3899, respectively; Figure 4A). Thus, the normalization of the glucose profile appeared to reflect a combination of reduction in fasting glucose, modification of glucose excursion and an altered rate of decay of circulating glucose (see below). Serum levels of glucose-stimulated insulin were simultaneously monitored in the same experiment. DIO mice displayed a severe hyperinsulinemia: the serum concentration increased more than 7-fold compared to SCD controls (*P* = 0.0003). In contrast, subchronic administration of 3 mg Boc5 induced a dose-dependent (*P* = 0.0007) reduction of overall insulin excursion by up to 85% (*P* = 0.0062) after an intraperitoneal (i.p.) glucose challenge (Figure 4B). This was accompanied by a significant and dose-dependent (*P = *0.0225) diminishment of pancreatic insulin content in DIO mice following 12 weeks of Boc5 treatment, a phenomenon that was not observed in pair-fed controls (Figure 4C). Further, despite equal energy consumption and submaximal weight loss, unlike 3 mg Boc5-treated DIO counterparts, pair-fed mice still exhibited deterioration of glucose tolerance (*P*<0.0001; Figure 4A) and hyperinsulinemia (*P*<0.0001; Figure 4B) during this period.

The data of Boc5 treatment on glucose homeostasis and hyperinsulinemia were suggestive of improvement in insulin sensitivity. To verify this, a separate set of insulin tolerance tests (ITT) experiments were carried out to measure the rate of glucose clearance in response to exogenous insulin. The overall glucose response in mice is typically consisting of an initial fall (attributable to insulin) followed by a rise (attributable to glucagon and other counter-regulatory hormones) in serum concentrations. As expected, the initial rate of glucose fall in response to 2 IU/kg recombinant human insulin (K_itt_) was 1.9-fold higher in lean than in obese mice (*P* = 0.0138); it was increased in a dose-dependent manner up to 1.8-fold in DIO mice receiving subchronic Boc5 (3 mg) administration (*P* = 0.0190). Pair-fed mice, however, showed a restoration trend but failed to reach the similar level seen in the 3 mg Boc5-treated group (Figure 4D).

### Effect on the pancreas

After 12 weeks of treatment, total pancreatic weight did not differ among lean, obese, Boc5-treated and pair-fed groups (data not shown). Histological examination revealed the frequent appearance of larger islets and microvesicles in islet cells of obese mice. Enlarged interlobular interspaces and lipid deposition were also found in some pancreatic specimens, but inflammatory cell infiltration was not obvious and only a few lymphocytes were observed in inter- or intra-lobular areas. Such alterations were greatly diminished following Boc5 treatment whereas the histological changes seen in pair-fed mouse pancreases remained notably significant (Figure S3).

**Figure 5:**
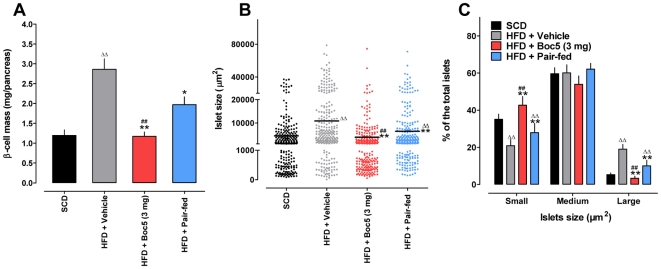
Effects of Boc5 on β-cell mass and islet size. (A) β-cell mass (6 sections per pancreas) was quantified using the Image-Pro Plus 6.0 software and calculated by multiplying the percentage of insulin-immunostained area by pancreatic weight. (B) The area of each islet was assessed by manually drawing around the islet perimeter with the measuring tool contained in the software. The total numbers of islets counted for each group were: 262, 299, 280 and 263, respectively. Y-Axis represents the islet size that was divided into three fractions: small, medium and large. (C) Distribution of small, medium and large islets as a percentage of the total islets in obese mice with or without 3 mg Boc5 treatment. Values represent mean±SEM. ^ΔΔ^ *P*<0.01 compared with SCD group; ^*^ *P*<0.05 and ^**^ *P*<0.01 compared with HFD group; ^##^ *P*<0.01 compared with pair-fed group that received an equal amount of food as 3 mg Boc5-treated mice.

Absolute β-cell mass was decreased by 59% in the 3 mg Boc5-treated group (1.17±0.27 mg/pancreas, *P* = 0.000146) and 31% in the pair-fed group (1.97±0.47 mg/pancreas, *P* = 0.005878), respectively, compared to the obese group (2.86±0.64 mg/pancreas). The difference between Boc5 (3 mg) treated obese and lean mice was negligible (*P* = 0.8842; Figure 5A). The changes in β-cell mass were consistent with both fasting serum insulin levels and pancreatic insulin contents measured simultaneously (Figures 4B and 4C).

We also examined alterations in islet size following Boc5 treatment. Islets were divided into three categories: small (<1000 µm^2^), medium (1000–20000 µm^2^) and large (>20000 µm^2^). It was found that obese mice receiving Boc5 had a decreased islet size compared to that of untreated ones (3511±350.8 µm^2^ *vs*. 10630±806.0 µm^2^, *P*<0.0001; Figure 5B). This was accompanied by a marked increase in small islets in Boc5-treated mice as opposed to obese controls: the percentage of large islets was 3.33% *vs*. 19.02% (*P* = 0.0002) while that of small islets was 42.72% *vs*. 20.85%, respectively (*P* = 0.0035; Figure 5C). It appears that the reduction of β-cell mass induced by Boc5 might be resulted from such a dramatic rise in the number of small islets (Figure S4).

### Effects on liver/muscle weight, triglyceride content and liver morphology

**Figure 6:**
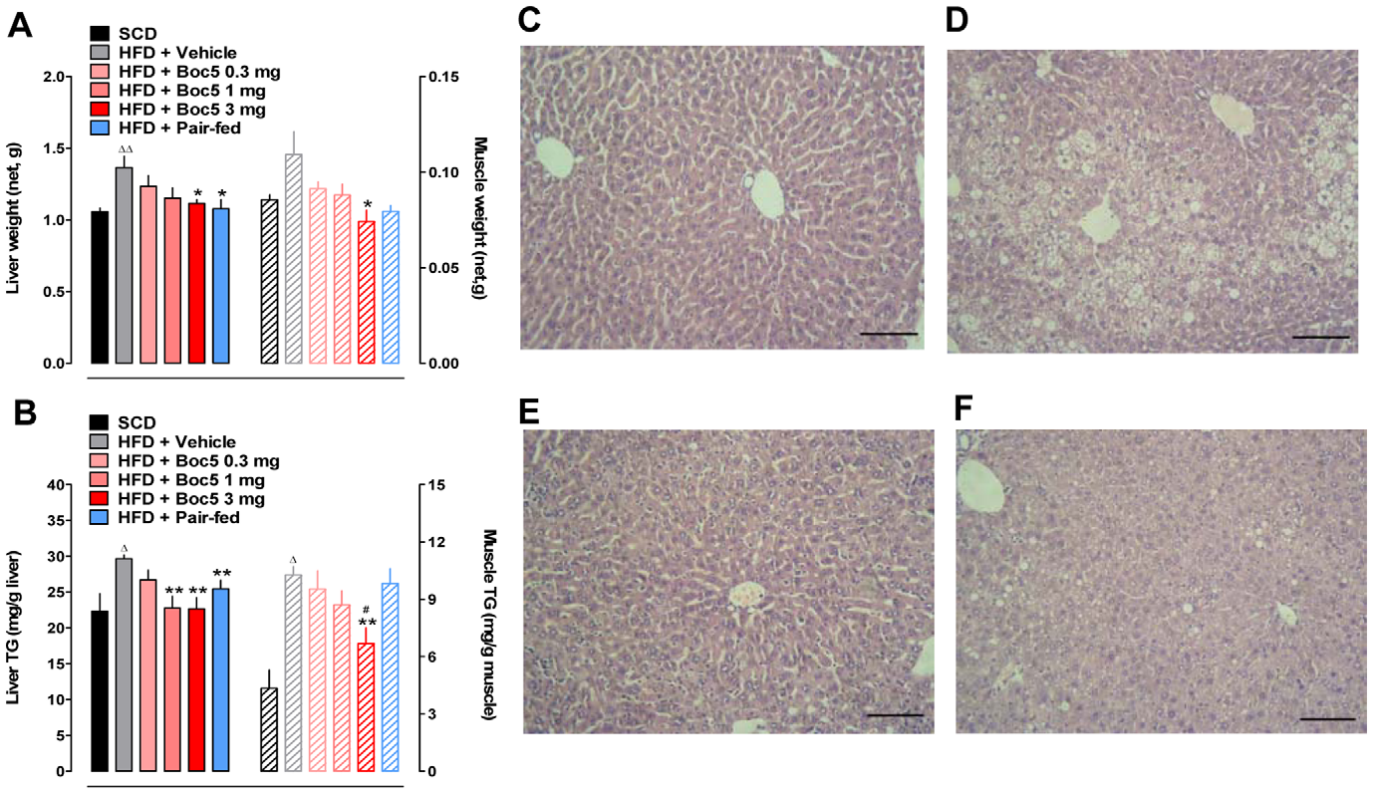
Effects of Boc5 on liver/muscle weight (A), triglyceride (TG) content (B) and liver morphology (C–F). The weights of liver and muscle from each treatment group and SCD control were determined after 12 weeks of treatment and the TG content examined following tissue lipid extraction. Values represent mean±SEM (n = 6–8 per group). ^Δ^ *P*<0.05 and ^ΔΔ^ *P*<0.01 compared with SCD group; ^*^ *P*<0.05 and ^**^ *P*<0.01 compared with HFD group; ^#^ *P*<0.05 compared with pair-fed group that received an equal amount of food as 3 mg Boc5-treated mice. Histological analysis was performed on the liver tissue from lean (C), obese (D), 3 mg Boc5 (E) and pair-fed (F) treated mice, respectively (n = 3 per group). Sections (5 µm) were stained with H&E and representative images (×200 original magnification) obtained after the 12-week observation period. Scale bars = 50 µm.

Compared with SCD, HFD feeding for 12 weeks significantly increased net liver weight by approximately 29.2% (*P = *0.0002; Figure 6A). Boc5 treatment led to a dose-dependent decrease in liver weights by 9.6%, 15.6% and 18.3% for 0.3 mg, 1 mg and 3 mg doses, respectively, among which the reduction induced by 3 mg of Boc5 was statistically significant (*P = *0.0213, compared to untreated obese mice). Surprisingly, similar reduction (about 23.4%, *P = *0.01797) was also observed in pair-fed animals, though their total adipose tissue weight was heavier than that of Boc5 (3 mg) treated group. There seemed to be a tendency of increased gastrocnemius muscle weight (+27.6%) in obese mice but the difference was not statistically meaningful in comparison with lean controls (*P* = 0.0639). Boc5 administration, other than pair-feeding, displayed a significant reduction of liver weight (*P* = 0.0473; Figure 6A).

Histological examination also confirmed these findings. Liver sections from DIO mice exhibited extensive intracellular vacuolization and significant lipid accumulation in both perivenular and periportal areas (Figure 6D). In contrast, only scattered small lipid droplets were detected in the liver from Boc5 (3 mg) treated animals (Figure 6E).

For further analysis, liver and muscle lipids were extracted for determination of triglyceride (TG) levels (Figure 6B). Compared with SCD controls, liver and muscle TG stores were elevated by 32.8% (*P* = 0.0278) and 139.5% (*P*<0.0001) in DIO mice, respectively. Boc5 dose-dependently reduced TG contents in these tissues by up to 23.3% (liver) and 35.0% (muscle), respectively, throughout the 12 weeks of treatment. The concentration of TG in the liver, rather than in the muscle, was significantly decreased in pair-fed mice in comparison with obese controls (*P* = 0.0044).

### Effect on blood chemistry

Recent evidence suggests that obesity and insulin resistance represent important risk factors for steatohepatitis [32]. In the present study, hepatocyte damage was assessed by examining serum enzyme activities of alanine aminotransferase (ALT) and aspartate aminotransferase (AST). Our data (Table 2) showed that ALT concentration was 2.5-fold higher in obese than in lean mice (*P* = 0.0008). Boc5 treatment resulted in a reduction at each dose studied (0.3, 1 and 3 mg), though there was no apparent dose-response relationship observed (47.9–57.1% decreases). Pair-feeding was also able to reduce ALT level by 45.2%, similar to the effect of 3 mg Boc5 (*P*>0.05). Serum AST concentration in DIO mice was trending higher than in SCD controls (*P* = 0.071), but only at the 3 mg dose, Boc5 evoked a significant decrease (*P* = 0.0003) in AST level.

**Table 2:** Effects of Boc5 on serum biomarkers examined at the end of the 12-week therapy.

		HFD				
	SCD	Vehicle	Boc5 0.3 mg	Boc5 1 mg	Boc5 3 mg	Pair-fed
**ALT (IU/L)**	22.71±10.78	56.58±25.84ΔΔ	29.50±15.05**	24.25±7.07**	28.29±5.32**	31.38±4.24**
**AST (IU/L)**	131.79±26.46	150.33±21.27	159.38±33.95	131.63±32.72	111.88±15.6**, #	136.25±20.24
**TC (mM)**	2.59±0.75	5.20±0.97ΔΔ	4.40±0.44*	3.43±0.56**	2.54±0.20**, ##	5.14±0.35
**TG (mM)**	1.02±0.37	1.44±0.32ΔΔ	1.46±0.18	1.21±0.21	0.70±0.20**, ##	1.45±0.26
**NEFA (mM)**	1.11±0.22	1.43±0.22ΔΔ	1.38±0.17	1.26±0.19	1.16±0.29*, ##	1.55±0.35
**LDL/HDL**	0.24±0.16	0.34±0.08Δ	0.31±0.06	0.23±0.06**	0.25±0.04**, #	0.24±0.06**

Values represent mean±SEM (n = 8–12 per group).

Δ *P*<0.05 and

ΔΔ *P*<0.01 *vs*. normal diet (SCD) group;

*P<0.05 and

**P<0.01 *vs*. high fat diet (HFD) group that received vehicle treatment;

# *P*<0.05 and

## *P*<0.01 *vs*. HFD group that was pair-fed an equal amount of food as 3 mg Boc5-treated mice.

Serum TG, total cholesterol (TC) and nonesterified fatty acid (NEFA) levels were markedly elevated in obese mice (*P* = 0.0049, *P*<0.0001 and *P* = 0.0008, respectively, compared to lean mice). Boc5 (3 mg) treatment, but not pair-feeding, reversed these metabolic parameters (*P*<0.0001, *P*<0.0001 and *P* = 0.0387, respectively, compared to obese mice), which became indistinguishable from that seen in SCD fed animals. In addition, measurement of circulating high-density lipoprotein (HDL) and low-density lipoprotein (LDL) elicited an unexpected but explainable [33], [34] result that both “good” (HDL) and “bad” (LDL) cholesterol values were increased after HFD feeding (data not shown). We therefore calculated the LDL/HDL ratio to analyze the magnitude of increases in the two lipoproteins among different treatment groups. As shown in Table 2, long-term exposure to HFD increased the LDL/HDL ratio by 45%, which was reduced to the normal range following Boc5 administration (1 and 3 mg) or diet suppression (*P* = 0.0019, *P* = 0.0036 and *P = *0.0038, respectively).

## Discussion

GLP-1 is recognized as an important endogenous regulator of glucose and lipid homeostasis. Efforts to utilize GLP-1 analogs or GLP-1R agonists in the treatment of T2DM and obesity have lasted for decades. The successful clinical use of Exendin-4 and Liraglutide [19] - injectable peptidic GLP-1R agonists and multiple ongoing human trials with other GLP-1 peptidomimetics all support the idea that GLP-1 analogs are probably the most beneficial therapeutic agents for T2DM in today's medical practices [20]. The major pharmaceutical hurdle for an effective oral treatment has been the difficulty to find a non-peptidic GLP-1R agonist with a long half-life [35]. Boc5 is one of the first such compounds that demonstrated an array of therapeutic actions in the treatment of diabetes and obesity in *db/db* mice [26], [27]. Boc5 efficiently induced a durable restoration of glycemic control and its other dose-dependent effects include reduction in food intake, slowing of gastric emptying, stimulation of insulin secretion and elevation in insulin sensitivity following 4 weeks of daily administration. It also decreased body weight of diabetic *db/db* mice but required a high dose (3 mg per day) [27]. In the present study, we employed a rodent model of DIO to overcome the shortcomings of previously used genetic model (the *db/db* mouse) and thus to provide data with more relevance to human diseases: obesity and T2DM [30]. DIO model can in part mimic human energy consumption patterns and gives the possibility of studying the pathogenesis of obesity and related diseases (*e.g.*, T2DM) and examining the consequences of therapeutic intervention [36], [37]. This model was originally introduced by Surwit *et al*. in 1988 [38] and has been shown to be most efficient in C57 mice compared with other strains [28], [29]. Therefore, this was the rationale of utilizing HFD fed C57 mice to advance our understanding on the pharmacological properties of Boc5 in diet-induced obesity and related metabolic disorders.

During the 12-week induction period with HFD, energy consumption of C57 mice was significantly increased and their body weight gained progressively, leading to moderate hyperglycemia, glucose intolerance and hyperinsulinemia. A pilot experiment was performed to determine the treatment regimen with Boc5, in which drastic weight loss was observed following daily dosing especially at 3 mg (data not shown). In order to avoid possible adverse consequences [39], [40], we applied an intermittent (tiw) dosing schedule to the present work. Our results clearly demonstrate that subchronic Boc5 treatment invoked marked suppression of food intake and sustained reduction of body weight in DIO mice with both 1 mg and 3 mg doses (Figures 1A, 1C and 1D). Although similar effects were seen in *db/db* mice, Boc5 at a daily dose of 1 mg failed to induce marked weight loss [27]. The anti-obesity action of Boc5 is in agreement with a recent study with Exendin-4 conducted in HFD fed C57 mice [41]. While the weight-lowering effect of Exendin-4 mainly occurred in the first week of treatment [41], Boc5 seemed to manifest its regulatory role in a more sustainable manner, *i.e.*, affecting the entire therapy period. As a result, a 30% loss from the initial body weight was achieved (Figure 1A) and maintained for at least 7 weeks after the cessation of Boc5 therapy (3 mg; Figure S5). This long-lasting benefit is consistent with that observed in rats following administration of GLP-1 [42]. Such a “memory effect” is probably attributable to its action on the transcription of key regulator genes controlling β-cell mass and function [41], [42]. BMI, an index reflecting severity of obesity derived from body weight and body length [43], was dose-dependently suppressed by Boc5 treatment, suggesting an ameliorated obese state in DIO mice (Figures 1B and 1C). Interestingly, despite equal energy intake, the pair-fed group showed body weight reductions only in the beginning of the 12-week treatment period and the effect was less pronounced than the Boc5-treated (3 mg, tiw) group. We presume that this phenomenon may have been caused by reduced body temperature and energy expenditure resulted from feeding suppression as a compensatory mechanism to conserve energy [44], [45].

For an ideal anti-obesity therapy, it is preferable that the weight loss stems predominantly from fat. In the present study, the remarkable decrease in body weight was accompanied by a dose-dependent reduction in fat as a percentage of body weight (Figure 1E). Analysis of carcasses from Boc5-treated DIO mice indicates that the body mass was preferentially lost from fat. On the other hand, despite the observed fat diminishment, such regulated loss invoked by Boc5 did not reach the extent to bring adverse consequences that often occurs in adipose tissue ablated mice [39], [40]. It is known that increase in adipocyte size due to fat storage (adipocyte hypertrophy) plays a key role in the formation of adipose tissue mass, and such conversion from small into large adipocytes is closely related to common health risks including hyperlipidemia, diabetes, hypertension and cardiovascular diseases [46]. Our histological examination suggests that increases of fat deposits in adipose tissue were accompanied by white and brown adipocyte hypertrophy in HFD fed obese mice (Figures 2A and 2B). In contrast, white adipocytes from Boc5-treated mice appeared to be smaller in size than that from vehicle-treated controls, an observation consistent with the low TG and NEFA levels in the circulation (Figures 2A and 2C, Table 2). Boc5 treatment also led to a reduction of lipid content in brown adipocytes (Figure 2B) thereby reflecting the adaptive status of BAT in regulating thermogenesis [47], [48].

Adipose tissue has a substantial influence on systemic metabolic homeostasis via its role as an endocrine organ capable of secreting diverse adipocytokines [49], [50]. The dose-dependent reduction of serum leptin level towards the normal range observed in Boc5-treated mice (Figure 1F) supports the existence of a correlation between circulating leptin and adiposity. This phenomenon was accompanied by simultaneous restoration of circulating adiponectin concentration to a level that is completely normal (Figure 1G). Such an altered secretion associated with changes in adiposity is suggestive of a potential role for adiponectin as an autocrine factor in WAT to modulate adipocyte size (secretion increases when fat mass is reduced but it decreases following fat deposition) [51]. Different from Boc5 treatment, caloric restriction did not affect adiposity significantly and circulating adipocytokines levels remained abnormal (Figures 1E–1G and Figure 2). These apparent differences imply that the regulatory role exerted by Boc5 (and hence, incretin mimetics in general) on lipid metabolism is independent of its inhibition on food intake.

Numerous studies have suggested that obesity is accompanied by several related metabolic defects in adipocytes concerning glucose or free fatty acid uptake, lipolytic activity and lipid oxidation [52]. Our results indicate that the basal glucose incorporation was significantly elevated in adipocytes isolated from obese mice while their response to insulin stimulation was severely impaired (Figures 3A and 3B). Although there are conflicting reports regarding the alteration in the basal glucose uptake, most previous findings demonstrate that insulin-induced glucose incorporation is suppressed in the obese state [53]–[55]. Meanwhile, we observed that the basal lipolytic activity was increased in obese mouse adipocytes that also displayed evident resistance to NA stimulation (Figures 3C and 3D). This finding is consistent with earlier results obtained from human or animal adipocytes [56], [57]. There is evidence suggesting that increased cAMP and tumor necrosis factor α (TNF-α) production in fat cells from obese individuals may be responsible for the enhancement of basal lipolysis [58], [59], whereas lipolytic resistance is probably due to decreased expression of β_2_-adrenoceptors [57] and hormone-sensitive lipase (HSL) capable of hydrolyzing TG [60]. Even if Boc5 treatment did not normalize basal and stimulated glucose uptake and lipolysis in our *ex vivo* experiments, the improvements were impressive (Figure 3), especially in the context that both GLP-1 and Exendin-4 only exhibited modest effects on fat cell (rat or human) metabolism when introduced directly to culture medium [55], [61]–[64]. Similar to the data described elsewhere [54], the role of caloric restriction (pair-feeding) in modifying fat cell lipogenesis and lipolysis was minimal (Figure 3), thereby further supporting the existence of an anorexia-independent pathway for Boc5 in modulating lipid metabolism.

It is well established in C57 mice that consumption of HFD results in both moderate hyperglycemia and progressive hyperinsulinemia, leading to eventual insulin resistance [30]. In the present study, we observed that fasting glucose levels in untreated obese mice were deteriorating with time while Boc5 could reverse such a tendency (Table 1). Combined with the dose-dependent normalization of IPGTT, glucose-stimulated insulin release and pancreatic insulin content (Figures 4A–4C), our data point to a Boc5-mediated glycemic control mechanism where restoration of insulin sensitivity as well as reduction of peripheral demand for insulin each plays its role. The increased rate of glucose clearance in response to a fixed (2 IU/kg) amount of insulin (Figure 4D) provides additional independent evidence for an insulin sensitizing effect of Boc5 in DIO mice. We know that β-cells adapt to situations of chronic fuel over-supply and insulin resistance by increasing their mass [65], which in our hands was elevated 2.4-fold in untreated obese mice (Figure 5A). Although a non-biased “systematic uniform random sampling” method [66] was not used in this study to estimate the β-cell area, our findings appear to be indicative of alterations in several parameters related to β-cell function after 12 weeks of Boc5 treatment: (i) complete normalization of β-cell mass; (ii) reduction in islet size; (iii) increase in small islets (Figure 5); and (iv) suppression of pancreatic apoptosis. The latter was achieved by a significant decrease of caspase 3/7 activities in the pancreases of treated mice (Figure S6). These morphological or biochemical changes are in agreement with the previous findings with Exendin-4 [67], [68], and may collectively be attributable to the functionality improvement reflected by enhanced insulin sensitivity, reduced insulin demand and optimized β-cell efficiency [69].

The subchronic (12 weeks) insulin-sensitizing effect of Boc5 observed here not only reproduces our earlier results generated in *db/db* mice [27], but is also consistent with the responses to chronic GLP-1R agonist treatments in humans [19] and rodents [70], [71] reported elsewhere. Moreover, since adipose tissue has a substantial influence on systemic glucose homeostasis through secretion of adipocytokines [49], [50], reduced adiposity is likely to contribute to the preservation of insulin sensitivity as well. Recent studies have defined adiponectin as an insulin sensitivity mediator which stimulates tissue fatty acid oxidation and inhibits hepatic glucose production by activating AMP-activated protein kinase [72]–[74]. Our results thus suggest that improvement of insulin resistance in DIO mice treated with Boc5 may be at least partially mediated by adiponectin. More importantly, unchanged hyperglycemia, hyperinsulinemia, pancreatic insulin content and ITT response in pair-fed mice clearly indicate an absence of effect by caloric restriction on insulin sensitivity, thereby implying a possible dissociation between anorexic and insulin-sensitizing effects of Boc5 [27]. Obviously, part of the glycemic benefits of Boc5 manifested in obesity is exerted through its potential regulation of adiposity.

A direct consequence of HFD consumption is increased fat deposition in both adipocyte stores and non-adipose tissues including liver and skeletal muscle [75]. Our data confirm that liver and muscle weight gain is associated with TG content enhancement and hepatocyte fat infiltration (Figure 6). In addition, serum ALT and AST concentrations (Table 2), as biomarkers of liver integrity, were either elevated or showed such a tendency in obese mice, suggesting that obesity and steatohepatitis co-exist in this animal model. Abolishment of liver/muscle fat accumulation and hepatocyte injury following Boc5 administration collaborates with the results obtained in *ob/ob* obese mice and T2DM patients receiving mid- or long-term Exendin-4 treatment (60 days and 3 years, respectively) [76], [77]. Although insulin resistance is likely to account for liver and muscle damage [75], [78]–[80], somewhat surprising is that the apparently less severe tissue fat infiltration in pair-fed mice occurred without concordant restoration in insulin sensitivity.

It should be noted that 12 weeks' Boc5 treatment was associated with remarkable improvements of several dyslipidemia-related circulating parameters such as elevated TC, TG and LDL/HDL ratio (Table 2). Although variations of these physiological measures are influenced by strain/race and diet ingredients [81], they are believed to be predictive of heart disease and atherosclerosis in the obese and diabetic populations [5], [18], [82]. Therefore, intervention of obesity with incretin mimetics may have the potential to reduce the morbidity of cardiovascular diseases. Since NEFA released from adipose tissue has been proposed as a link between obesity and insulin resistance [83], the reduction of NEFA level resulted from Boc5 therapy (Table 2) may therefore be one possible mechanism responsible for the restoration of insulin sensitivity in obese animals. This assumption is consistent with the low basal lipolytic activity detected in Boc5-treated obese mice, because circulating NEFA level is a main indicator of basal lipolysis [84]. There were no significant differences in TC, TG and NEFA levels between obese and pair-fed animals, indicating that caloric restriction alone is not sufficient to correct the state of dyslipidemia in obesity.

In conclusion, this study using a rodent DIO model has confirmed the glycemic control and weight loss properties of Boc5, reported previously in *db/db* mice. Applying an intermittent dosing protocol, subchronic Boc5 exhibited typical dose-responses in regulating food intake, adiposity and glucose homeostasis. It is also efficacious in treating multiple conditions associated with obesity such as dyslipidemia, adipocytokines dysregulation, adipocyte malfunction and liver injury. These findings suggest that Boc5 may produce metabolic benefits via an array of synergistic mechanisms. Further investigations are required to expand our knowledge on this class of non-peptidic GLP-1R agonists aiming at pharmacotherapies for obesity and related metabolic diseases.

## Materials and Methods

### Animals and diets

Eight-week old male C57BL/6J mice (18–20 g; Shanghai SLAC Laboratory Animals Co., Shanghai, China) were housed (two per cage) at 22.7±0.8°C in a 12∶12 h light:dark cycle. Animals were fed a HFD (D12492; 60% fat, 20% protein and 20% carbohydrate; 5.24 kcal/g) or a SCD (D12450B; 10% fat, 20% protein and 70% carbohydrate; 3.85 kcal/g) and watered *ad libitum*. Both diets were supplied by Research Diets (New Brunswick, NJ, USA). Animal experimentation was conducted in accordance with the regulations approved by the Animal Care and Use Committee, Shanghai Institute of Materia Medica, Chinese Academy of Sciences.

### Experimental design

Experimental animals were maintained on prescribed HFD for 12 weeks and then randomly assigned into 5 treatment groups (n≥6 per group) with matched body weight. They were injected (i.p.) three times a week, in the mornings of each Monday, Wednesday and Friday, with 0 (vehicle control), 0.3, 1 or 3 mg Boc5 (1% DMSO, 20% PEG400 in saline, pH 7.4, 0.5 ml) for 12 weeks. To account for secondary effects that might be due to a drug-induced reduction in food intake, a pair-fed control group was presented with the same quantity of food as the Boc5 3 mg treated group. A further comparator group of mice eating SCD (n = 15) was used to index responses to normal values.

Body weight and food intake were monitored daily. Blood samples were taken from the tail vein for overnight fasting blood glucose assessment (using a Freestyle Mini™ blood glucose monitoring system; Abbott Diabetes Care Inc., Alameda, CA, USA) and IPGTT every 3 weeks. Body length was measured at intervals of 6 weeks for calculation of BMI according to the formula reported elsewhere [43]. Before and after treatment, ITT were carried out to estimate insulin sensitivity.

At the end of the study, blood samples were collected and sera separated subsequently for further analyses. Mice were sacrificed to dissect and weigh white fat pads (mesenteric, gonadal, retroperitoneal and inguinal) and BAT according to various anatomical landmarks [85]. Weights were summed and expressed as a fraction of total body weight. Fresh gonadal fat pads were used to assess adipocyte glucose uptake and lipolysis. Pancreas, liver and skeletal muscle (gastrocnemius) were isolated and frozen by liquid nitrogen for insulin or lipid content measurement. Inguinal fat pads, BAT, liver and pancreas were fixed in 10% neutral buffered formalin for histological or immunohistochemical observation. The carcasses were stored at −80°C for total body fat evaluation.

### Glucose homeostasis

In IPGTT, each animal was fasted overnight, challenged i.p. with 2 g/kg D-glucose (Sigma-Aldrich, St. Louis, MO, USA) followed by serial assessment of blood glucose up to 120 min. Blood samples were collected at 0, 15, 30 and 60 min, respectively, for subsequent serum insulin analysis using the ELISA kit (EXRMI-13K) supplied by Linco Research (St. Charles, MO, USA).

Similar to those developed for clinical use [86], ITT comprised a 2 IU/kg i.p. challenge with recombinant human insulin (Humulin® R, Lilly Egypt, Giza, Egypt) in 4-hour fasted mice followed by glucose sampling at 30 min intervals. The rate of insulin-mediated fall in blood glucose was quantified to derive an initial rate of decay (K_itt_) as described previously [27].

### Immunohistochemical examination of the pancreas

Measurement of islet size and β-cell mass was performed using methodologies described elsewhere with some modifications [67], [87]. Mice were anesthetized followed by cervical dislocation. Whole explanted pancreases from six mice per treatment group (SCD, HFD, 3 mg Boc5 and pair-fed) were rapidly removed, cleared of fat and lymph nodes, weighed, fixed with 10% formalin overnight in a flattened position and embedded in paraffin. Ten consecutive sections (5 µm) of each tissue block, separated by at least 200 µm, were made and immunostained for insulin or glucagon. The sections were de-waxed, re-hydrated, and digested with 0.05% trypsinase at 37°C for 15 min, and blocked by 10% goat serum (blocking solution) in phosphate buffered saline (PBS) for 3 h at room temperature (RT). Incubation of samples overnight at 4°C with polyclonal guinea pig anti-swine insulin (1∶100; DakoCytomation, Carpinteria, CA, USA) or rabbit anti-human glucagon (1∶200; Cell Signaling Technology, Danvers, MA, USA) antibodies was then conducted in the blocking solution at RT, followed by twice washing in PBS before reacting with 1∶200 either Alexa Fluor® 488 conjugated goat anti-guinea pig or Alexa Fluor® 568 conjugated goat anti-rabbit IgG antibodies (Molecular Probes, Eugene, OR, USA) in the blocking solution for 2 h at 37°C. Following two washes with PBS, DAPI (4,6-diamidino-2-phenylindole, 0.5 mg/ml in PBS) was added onto the slides that were washed again after 10 min. They were then mounted with Slowfade Antifade Medium (Invitrogen, Carlsbad, CA, USA), examined and recorded blindly by two investigators using an epiflourescence microscope (×100; Olympus, Tokyo, Japan) with a grey level CCD (charge coupled device) camera (Olympus).

Histomorphometric measurements were performed with Image-Pro Plus 6.0 software (Media Cybemetics, Silver Spring, MD, USA). Six representative sections of each pancreas were chosen for analysis; each of them was examined for the presence and quantity of β-cells immunostained positively for insulin, and a minimum of 120 β-cell images per pancreas were analyzed. The cross-sectional area of β-cells was determined by quantitation of the area occupied by fluorescently labeled β-cells (green; all fields with β-cells present, a minimum of 20 images) and the cross-sectional area of all tissue visualized by DAPI (blue fluorescence). β-cell mass was estimated as the product of the total cross-sectional β-cell area over total tissue area and the weight of the pancreas before fixation. Islet area was traced manually and measured using the above software to assess islet size distribution. The total numbers of islets counted for SCD, HFD, 3 mg Boc5 and pair-fed groups were 262, 299, 280 and 263, respectively, and the median islet area for each group was calculated using the Image-Pro software.

### Glucose uptake and lipolytic activities in adipocytes

#### Isolation of adipocytes

Adipocytes were prepared as previously described [88]–[90]. Briefly, gonadal adipose tissue was dissected out, cut into small fragments and digested with 1 mg/ml collagenase Type I (Gibco/Invitrogen, Grand Island, NY, USA) in Krebs' Ringer phosphate (KRP) buffer (pH 7.4) with 4% (glucose uptake) bovine serum albumin (BSA; Sigma-Aldrich) or in KRB buffer with 25 mM Hepes (2-[4-(2-Hydroxyethyl)-1-piperazinyl]ethanesulfonic acid; Amresco, Solon, OH, USA), 5 mM glucose (Sigma-Aldrich) and 3% (lipolysis) BSA in a water bath at 37°C for 60 min. The isolated adipocytes were subsequently filtered through a nylon mesh and washed three times with KRP buffer containing 0.1% (glucose uptake) BSA or KPB buffer containing 1% (lipolysis) BSA. They were then counted, diluted in KRP buffer with 2% (glucose uptake) BSA or in KRB buffer with 3% (lipolysis) BSA and immediately used for glucose uptake or lipolysis experiments.

#### D-[3-^3^H]glucose uptake

Glucose uptake (as an index of lipogenesis) was measured by incorporation of D-[3-^3^H]glucose (PerkinElmer, Boston, MA, USA) into adipocyte lipids as described elsewhere [91], [92]. In brief, the adipocytes were incubated in KRB buffer with 2% BSA (pH 7.4) containing D-[3-^3^H]glucose (5×10^6^ dpm/ml), unlabelled glucose (1 µM) and different concentrations of insulin (0.3–10000 µIU/ml) to give a final volume of 0.5 ml in a shaking water bath at 37°C. The incubation was stopped by rapidly chilling on ice and adding 50 µl H_2_SO_4_ (6 M) after 2 h. Each vial was then left overnight with 1.5 ml of toluene-based scintillation liquid including toluene (Shanghai Chemical Reagents Co, Ltd., Shanghai, China), 2,5-diphenyloxazole (PPO; Alfa Aesar, Ward Hill, MA, USA) and 1,4 bis (4-methyl-5phenyl-2-oxazoyl) benzene (POPOP; Acros Organics, Morris Plains, NJ, USA) at RT. The incorporation of the radiolabeled glucose into the adipocyte lipids was determined by liquid scintillation counting on the MicroBeta scintillation counter (PerkinElmer).

#### Lipolysis

Lipolysis experiments were performed as reported in the literature [88] and glycerol release was measured as an index of lipolysis [93]. Briefly, adipocytes, in a final concentration of approximately 1.5×10^6^ cells/ml, were incubated for 20 min in a shaking water bath at 37°C in the presence of 0.1 IU/ml adenosine deaminase (ADA; Roche Diagnostics GmbH, Mannheim, Germany). Thereafter, 3, 30 or 300 nM of NA (Sigma-Aldrich) was added to the adipocytes after a 5-minute preincubation period. The incubation was stopped 60 min after the addition of NA. The glycerol release was measured essentially according to the protocol reported by Laurell *et al.* [93].

### Total body fat determination

Total body fat was measured as described by others [94], [95]. In brief, frozen carcasses were weighed (W_1_) and autoclaved in individual sealed beakers with distilled water (20% weight of the carcasses) for 2.5 h at 120°C. The contents were weighed (W_2_) followed by homogenizing into a paste. The latter (10 g) was lyophilized, weighed (W_3_) and powdered. Chloroform:methanol (2∶1) solution (20 ml) was used to extract total lipid in 1 g of the above powder at 40°C overnight. The extraction was filtered and adjusted to a constant volume (50 ml) with the chloroform:methanol solution. Then 10 ml of the filtrate were dried by rotary evaporation and the residual fat was weighed (W_4_). The body fat percentage was calculated with the formula below:
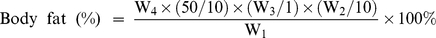



### Histology of liver and adipose tissues

Liver and adipose (WAT and BAT) tissues (n = 3) from each group (SCD, HFD, 3 mg Boc5 and pair-fed) were rapidly removed, fixed in 10% formalin overnight and embedded in paraffin. The same region of the tissue was used for all animals to minimize sampling variation that resulted from differences in cell size and anatomical location. Tissue blocks were sectioned 5 µm thick and stained with haematoxylin and eosin (H&E). Stained slides were viewed blindly and independently by two investigators under a microscope (Olympus) at ×200 magnification and images were captured by an Olympus C4000 zoom digital camera (Olympus). The size of the inguinal adipocyte was estimated using Image-Pro Plus 6.0 software (Media Cybernetics). Representative photomicrographs of the liver and BAT from each group were examined without statistical analysis. For white adipocyte size determination, multiple sections (separated by 70–80 µm each) were obtained from inguinal fat pads. Five representative sections of each mouse were analyzed for cell size and number using the software. For each animal, at least 10 fields (representing approximately 500 adipocytes collectively) were investigated as described in the literature [96].

### Determination of triglyceride contents in the liver and muscle

Hepatic and muscular TG levels were measured following the method of Folch *et al.* [97] with minor modifications. Briefly, approximately 100 mg of tissue was weighed and homogenized (17∶1, v/wt) in ice-cold chloroform:methanol (2∶1) solution for 1 min. The homogenates were filtered through Whatman No. 1 filter paper (Whatman Lab Sales Ltd., Maidstone, Kent, UK), which were subsequently washed (1.5∶1, v/wt) by chloroform:methanol solution twice. The filtrates were mixed with 0.2 volume of 0.9% NaCl solution and vortexed vigorously for 30 sec. The suspensions were then centrifuged at 2500–2700 rpm for 3 min at RT. The chloroform-methanol layer was removed, placed in a new glass tube and evaporated to dryness under vacuum in a rotary evaporator (50–55°C, 200 mBar). The lipid residue was resuspended in 0.5 ml 95% ethanol, and the TG concentration was determined enzymatically using the TG assay kit from Roche Diagnostics.

### Blood chemistry

The terminal blood samples were taken from orbital sinus for determination of fasting serum insulin, leptin and adiponectin levels using respective ELISA kits (EXRMI-13K, EZML-82K and EZMADP-60K) supplied by Linco Research.

Serum TG, TC, NEFA, HDL, LDL, ALT and AST were assayed on a Hitachi 7060 Automatic Analyzer (Tokyo, Japan) with kits from Roche Diagnostics and Wako Pure Chemical Industries (for NEFA; Osaka, Japan).

### Data analysis and statistical assessment

Dose- and concentration-responses were analyzed using Prism version 5 software (GraphPad, San Diego, CA, USA) to fit 4-parameter sigmoid functions. General effects were tested using 1-way ANOVA. Except where noted otherwise, pair-wise comparisons were performed using Dunnett's test for multiple comparisons, and *t*-test for simple pairs (unpaired *t*-test, GraphPad). Data throughout are stated as mean±SEM unless otherwise specified. Two-tailed significance was tested at α = 0.05. Where possible, experiments were designed with a sample size calculated from preliminary data to yield a power of B = 0.8.

## Supporting Information

Figure S1 Fat distribution following 12-week induction with a high fat diet (HFD). Both white and brown fat mass in normal C57 mice were significantly increased as a percentage of body weight following 12-week exposure to HFD (n = 9). Control animals received a standard chow diet (SCD; n = 8). Values represent mean±SEM. ^Δ^ *P*<0.05 and ^ΔΔ^ *P*<0.01 compared with SCD group. WAT, white adipose tissue; BAT, brown adipose tissue.
(0.24 MB TIF)

Figure S2 Normalization of glycemic control by Boc5. Subchronic Boc5 treatment (3 mg, tiw) progressively improved the area-under-curve values (AUC_120_) of intraperitoneal glucose tolerance tests (IPGTTs) carried out every three weeks. Control animals received a standard chow (SCD) or a high fat (HFD) diet with vehicle intervention. Values represent mean±SEM (n = 6 per group). ^ΔΔ^ *P*<0.01 compared with SCD group; ^**^ *P*<0.01 compared with HFD group.
(0.30 MB TIF)

Figure S3 Morphological examination of pancreases following 12-week Boc5 therapy. Treatment with Boc5 (3 mg, tiw) for 12 weeks markedly improved the pancreatic injuries induced by high fat diet (HFD). Sections (5 μm) of pancreatic tissue from control and Boc5-treated groups (n = 3 per group) were stained with H&E and representative histological images (×200 original magnification) were obtained. (A) No histopathological changes were noted in standard chow diet (SCD) group. (B) In HFD group, larger islets and frequent microvesicles in islet cells were observed. Enlarged interlobular interspaces and lipid deposition were also found in some pancreatic specimens. Inflammatory cell infiltration was not obvious and only a few lymphocytes were observed in inter- or intra-lobular areas. (C) In Boc5-treated mice, the alterations induced by HFD were mild. (D) The histological changes were also seen in pair-fed mouse pancreases without notable improvement. Scale bars = 50 μm.
(3.24 MB TIF)

Figure S4 Immunohistochemical analysis of pancreatic islets after 12-week Boc5 administration. Subchronic Boc5 treatment resulted in a dramatic rise in the number of small islets in the pancreases of obese mice. Red immunofluorescence staining indicates glucagon-producing cells and that of green shows insulin-producing cells. Blue fluorescence is the nuclear staining and I/G is the merge of the three. Images (×100 original magnification) were obtained from lean (SCD), obese (HFD), Boc5 (3 mg) and pair-fed treated mouse pancreases, respectively, following a 12-week therapeutic regimen.
(8.27 MB TIF)

Figure S5 Sustained weight loss after the cessation of subchronic Boc5 treatment. Except for standard chow diet (SCD) fed mice, all other four groups received high fat diet (HFD) without Boc5 or vehicle intervention. SCD+HFD group was served as DIO control whereas HFD+Pair-fed group was given an equal amount of food received by HFD+Boc5 3 mg group. Values represent mean±SEM (n = 8-10 per group). ^Δ^ *P*<0.05 and ^ΔΔ^ *P*<0.01 compared with SCD group; ^*^ *P*<0.05 and ^**^ *P*<0.01 compared with HFD group; ^#^ *P*<0.05 and ^##^ *P*<0.01; ^$^ *P*<0.05 and ^$$^ *P*<0.01 compared with SCD+HFD group.
(0.38 MB TIF)

Figure S6 Effect of Boc5 on Caspase 3/7 activity in the pancreas. Treatment with Boc5 (3 mg, tiw) for 12 weeks significantly reduced the pancreatic Caspase 3/7 activity of obese mice. The enzymatic activity was measured using a commercial Caspase 3/7 Glo assay kit from Promega (Madison, WI, USA). Relative luminescence unit (RLU) was normalized by the protein content of the pancreas. Control animals received a standard chow (SCD) or a high fat (HFD) diet with vehicle intervention. Values represent mean±SEM (n = 8 per group). ^ΔΔ^ *P*<0.01 compared with SCD group; ^**^ *P*<0.01 compared with HFD group.
(0.25 MB TIF)
