# Supplementary figures and images for: Reversal of Obesity and Insulin Resistance by a Non-Peptidic Glucagon-Like Peptide-1 Receptor Agonist in Diet-Induced Obese Mice
Source: PLoS One. 2010 Dec 3;5(12):e14205. doi: 10.1371/journal.pone.0014205 (PMC2997064; doi:10.1371/journal.pone.0014205)

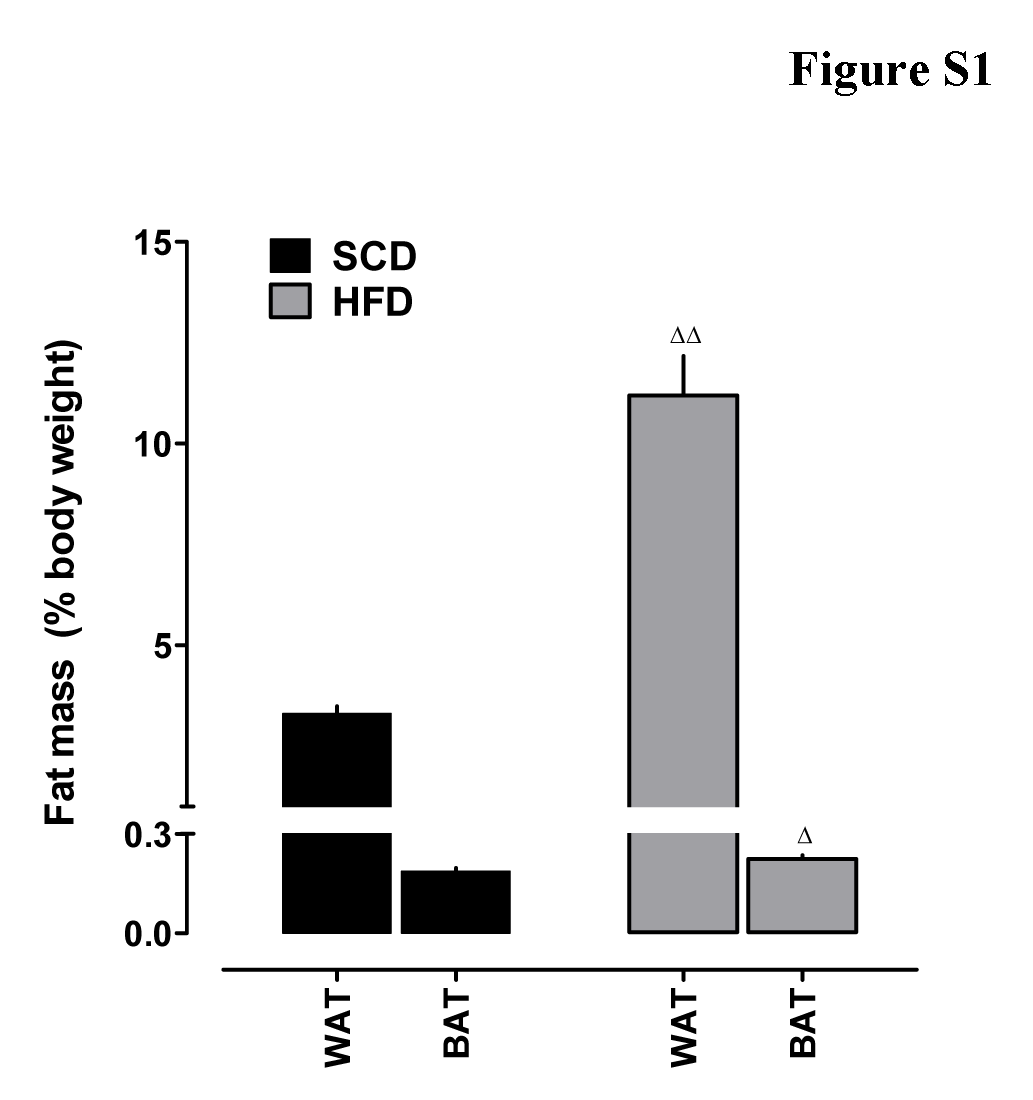

Supplement: Figure S1 — Fat distribution following 12-week induction with a high fat diet (HFD). Both white and brown fat mass in normal C57 mice were significantly increased as a percentage of body weight following 12-week exposure to HFD (n = 9). Control animals received a standard chow diet (SCD; n = 8). Values represent mean±SEM. Δ P<0.05 and ΔΔ P<0.01 compared with SCD group. WAT, white adipose tissue; BAT, brown adipose tissue. (0.24 MB TIF) [file pone.0014205.s001.tif]

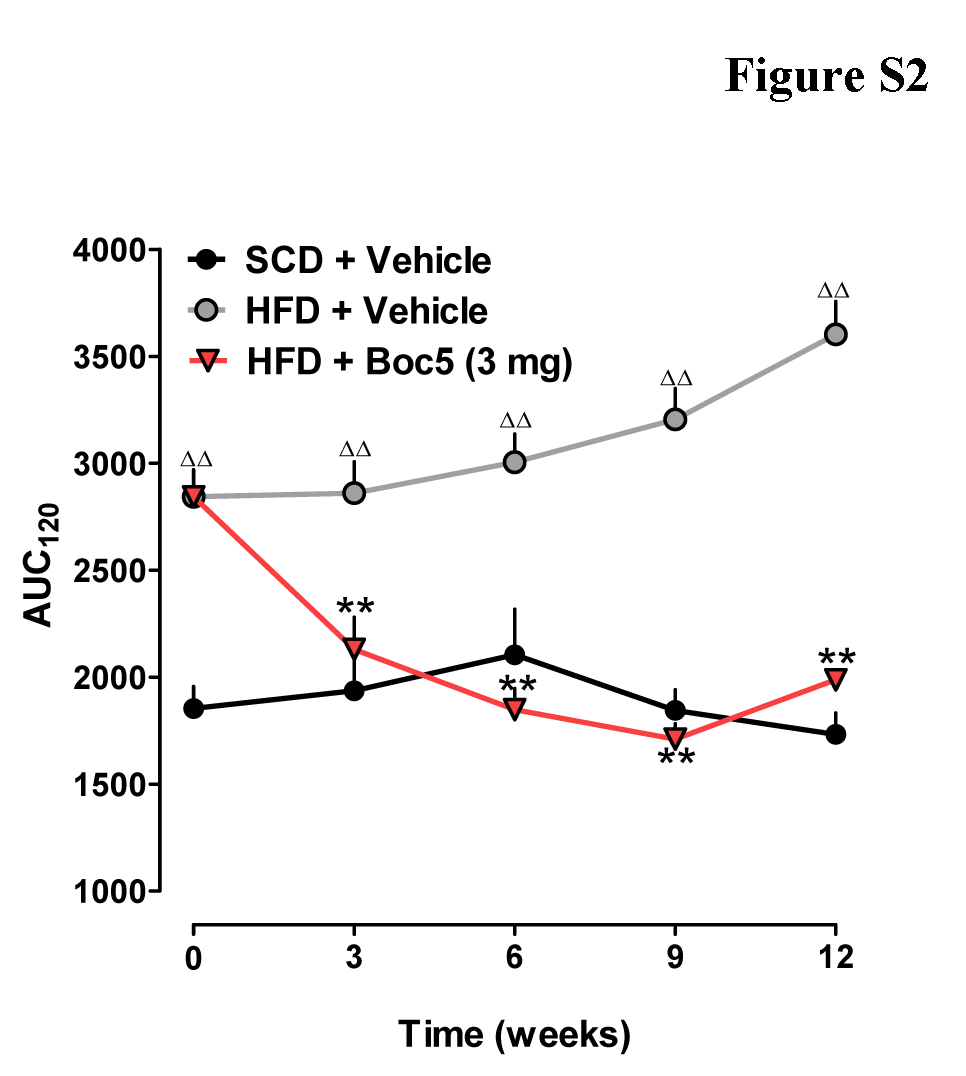

Supplement: Figure S2 — Normalization of glycemic control by Boc5. Subchronic Boc5 treatment (3 mg, tiw) progressively improved the area-under-curve values (AUC120) of intraperitoneal glucose tolerance tests (IPGTTs) carried out every three weeks. Control animals received a standard chow (SCD) or a high fat (HFD) diet with vehicle intervention. Values represent mean±SEM (n = 6 per group). ΔΔ P<0.01 compared with SCD group; ** P<0.01 compared with HFD group. (0.30 MB TIF) [file pone.0014205.s002.tif]

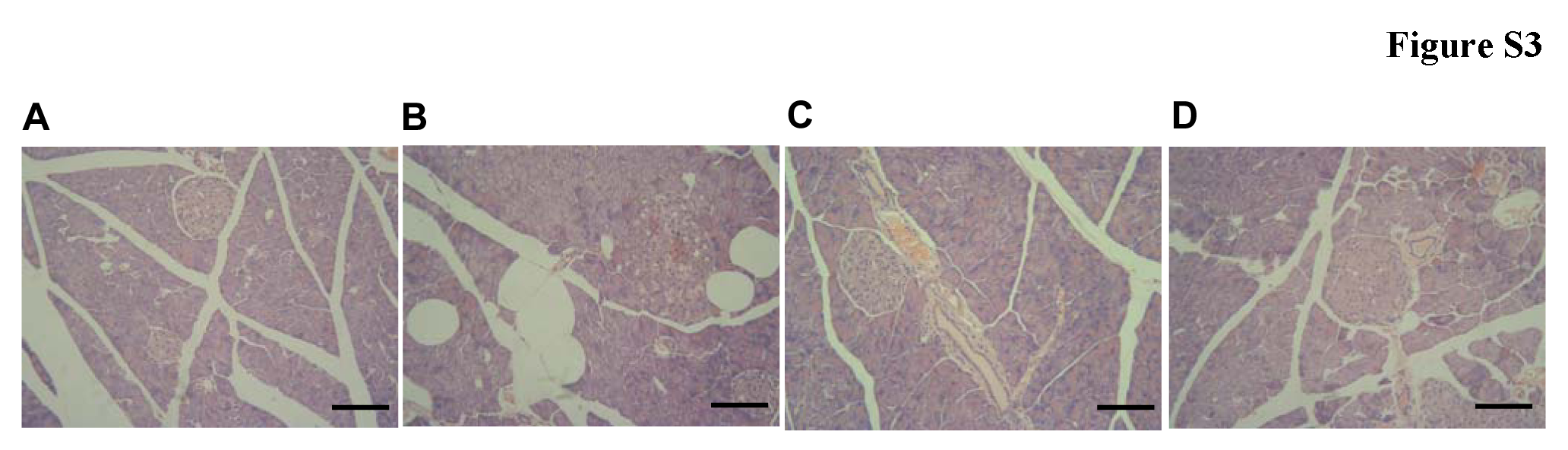

Supplement: Figure S3 — Morphological examination of pancreases following 12-week Boc5 therapy. Treatment with Boc5 (3 mg, tiw) for 12 weeks markedly improved the pancreatic injuries induced by high fat diet (HFD). Sections (5 μm) of pancreatic tissue from control and Boc5-treated groups (n = 3 per group) were stained with H&E and representative histological images (×200 original magnification) were obtained. (A) No histopathological changes were noted in standard chow diet (SCD) group. (B) In HFD group, larger islets and frequent microvesicles in islet cells were observed. Enlarged interlobular interspaces and lipid deposition were also found in some pancreatic specimens. Inflammatory cell infiltration was not obvious and only a few lymphocytes were observed in inter- or intra-lobular areas. (C) In Boc5-treated mice, the alterations induced by HFD were mild. (D) The histological changes were also seen in pair-fed mouse pancreases without notable improvement. Scale bars = 50 μm. (3.24 MB TIF) [file pone.0014205.s003.tif]

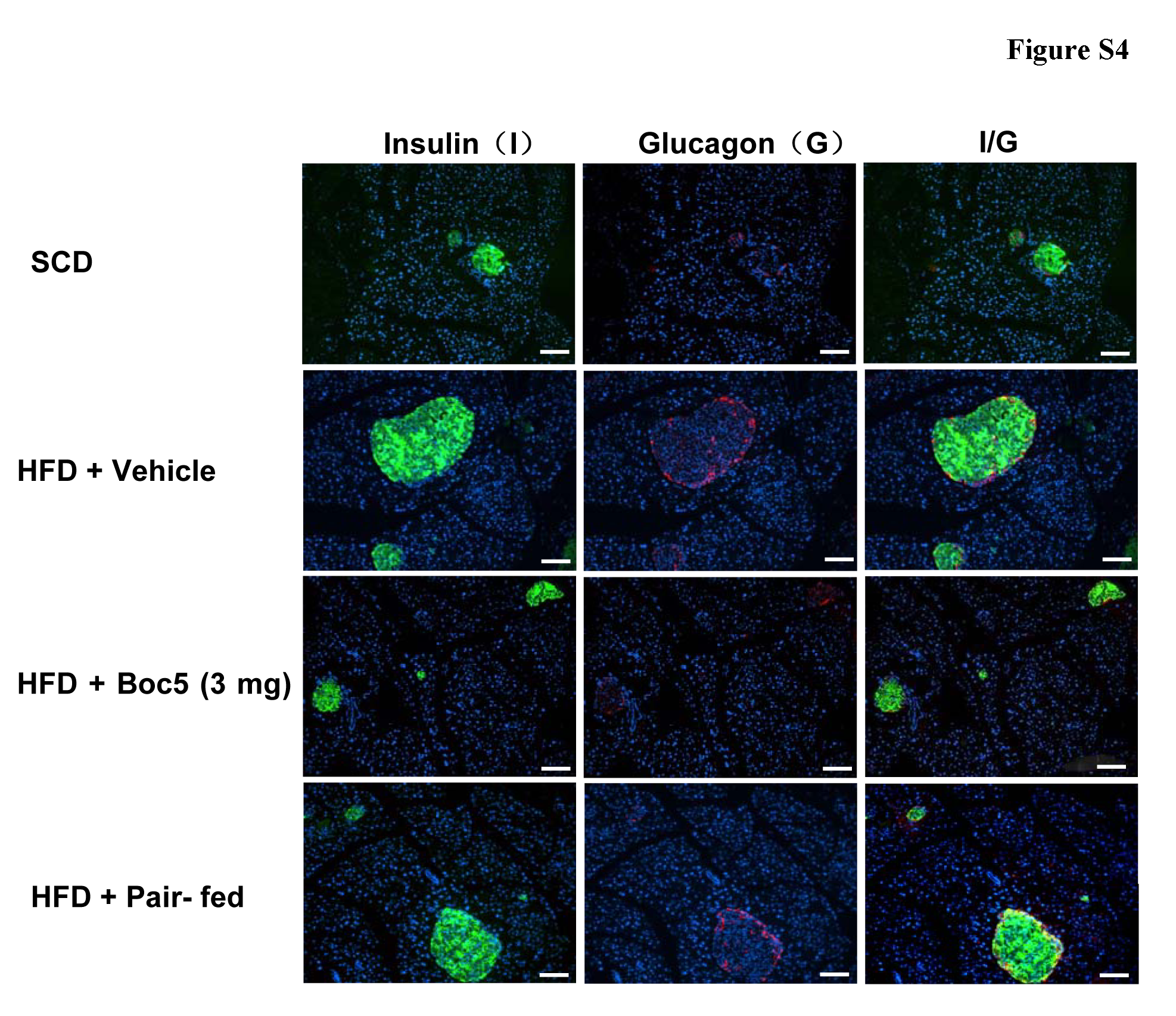

Supplement: Figure S4 — Immunohistochemical analysis of pancreatic islets after 12-week Boc5 administration. Subchronic Boc5 treatment resulted in a dramatic rise in the number of small islets in the pancreases of obese mice. Red immunofluorescence staining indicates glucagon-producing cells and that of green shows insulin-producing cells. Blue fluorescence is the nuclear staining and I/G is the merge of the three. Images (×100 original magnification) were obtained from lean (SCD), obese (HFD), Boc5 (3 mg) and pair-fed treated mouse pancreases, respectively, following a 12-week therapeutic regimen. (8.27 MB TIF) [file pone.0014205.s004.tif]

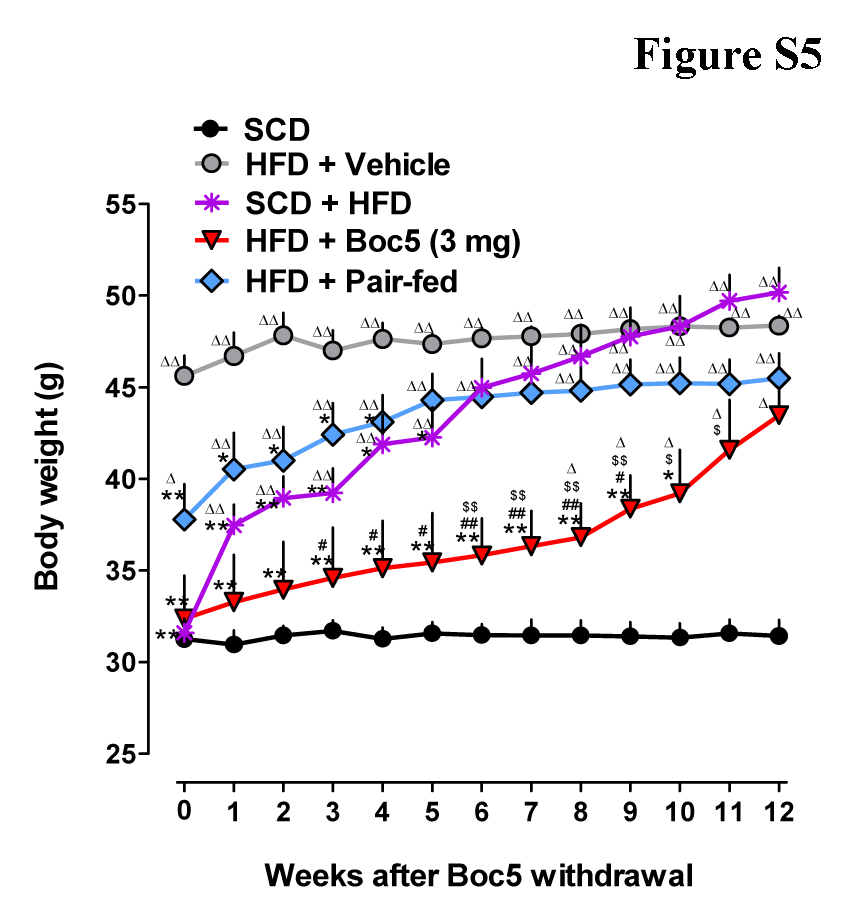

Supplement: Figure S5 — Sustained weight loss after the cessation of subchronic Boc5 treatment. Except for standard chow diet (SCD) fed mice, all other four groups received high fat diet (HFD) without Boc5 or vehicle intervention. SCD+HFD group was served as DIO control whereas HFD+Pair-fed group was given an equal amount of food received by HFD+Boc5 3 mg group. Values represent mean±SEM (n = 8-10 per group). Δ P<0.05 and ΔΔ P<0.01 compared with SCD group; * P<0.05 and ** P<0.01 compared with HFD group; # P<0.05 and ## P<0.01; $ P<0.05 and $$ P<0.01 compared with SCD+HFD group. (0.38 MB TIF) [file pone.0014205.s005.tif]

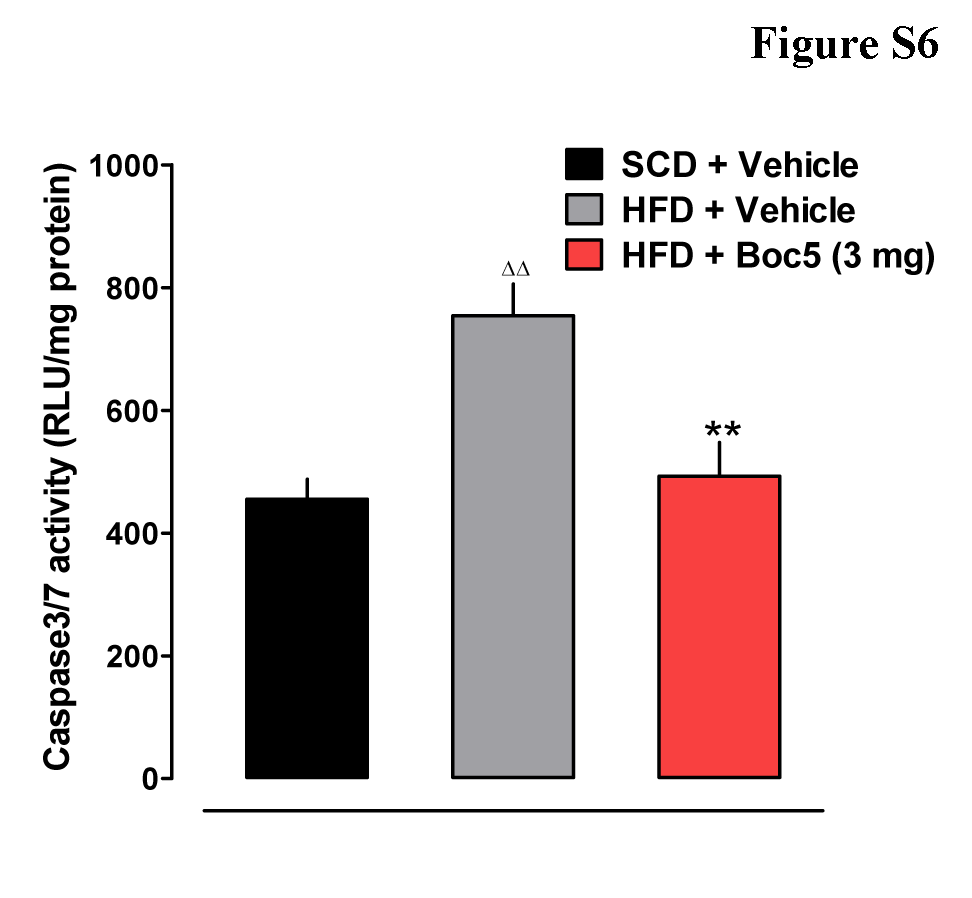

Supplement: Figure S6 — Effect of Boc5 on Caspase 3/7 activity in the pancreas. Treatment with Boc5 (3 mg, tiw) for 12 weeks significantly reduced the pancreatic Caspase 3/7 activity of obese mice. The enzymatic activity was measured using a commercial Caspase 3/7 Glo assay kit from Promega (Madison, WI, USA). Relative luminescence unit (RLU) was normalized by the protein content of the pancreas. Control animals received a standard chow (SCD) or a high fat (HFD) diet with vehicle intervention. Values represent mean±SEM (n = 8 per group). ΔΔ P<0.01 compared with SCD group; ** P<0.01 compared with HFD group. (0.25 MB TIF) [file pone.0014205.s006.tif]
